# The Internal Anatomy and Water Current System of Cambrian Archaeocyaths of South China

**DOI:** 10.3390/life14020167

**Published:** 2024-01-23

**Authors:** Jiayue Wang, Baopeng Song, Yue Liang, Kun Liang, Zhifei Zhang

**Affiliations:** 1 State Key Laboratory of Continental Dynamics, Shaanxi Key Laboratory of Early Life and Environments, Department of Geology, Northwest University, Xi’an 710069, China; wangjiayue@stumail.nwu.edu.cn (J.W.); baopeng_nwu@stumail.nwu.edu.cn (B.S.); 2 State Key Laboratory of Palaeobiology and Stratigraphy, Nanjing Institute of Geology and Palaeontology, Chinese Academy of Sciences, Nanjing 210008, China; kliang@nigpas.ac.cn

**Keywords:** archaeocyaths, morphology, anatomy, water current system, lower Cambrian

## Abstract

Archaeocyaths are a group of extinct filter feeders that flourished in the early Cambrian period and occupied an important position in the evolution of basal fauna and the early marine ecosystem. However, the detailed morphological and anatomical information of this group are still unclear due to insufficient fossil material and limited experimental analyses. Here, we report exquisitely preserved phosphatized archaeocyathan fossil cups, ca. 515 million years old, from the top of the Shuijingtuo Formation (Series 2, Stage 3) and the Xiannüdong Formation (Series 2, Stage 3) of the Yangtze Platform, South China. Detailed observation of their external morphology via scanning electron microscopy (SEM) and micro-computed tomography (Micro-CT) analysis revealed detailed information of their internal structure. They have a typical double-walled cup, with the perforated inner and outer walls concentrically distributed, but the structure between the two walls differs. The inverted cone-shaped cups have radially distributed septa between the walls. Perforated septa connect the two walls. The low and columnar cups have canals between the two walls, forming the network. These pores and cavities constitute an important component of the water current system (pumping and filtering water with a network of canals and chambers) and influence the process of filtration in the cup. In comparison to traditional thin-section analysis, the combination of SEM and Micro-CT analysis on phosphatized archaeocyaths presented in this study further explored the detailed internal structure and finely reconstructed the microscopic overall morphology and anatomy, which provide important information to help us understand the systematic taxonomy, anatomy, and morphology of archaeocyaths during the Cambrian period.

## 1. Introduction

As a member of basal animals, archaeocyaths occurred in the Terreneuvian Epoch and rapidly radiated during the Cambrian Series 2, witnessing the first occurrence of Small Shelly Fossils (SSFs) as well as the peak of the opening act of the Cambrian explosion [1,2,3,4,5]. They appeared as the first animals to secrete thick carbonate skeletons and occurred in all continents in the early Cambrian period, with the most well-preserved and best-known examples from Siberia, Southern Australia, North America, and South China [6,7,8,9,10,11,12,13,14,15,16]. In South China, archaeocyathan fossils mostly occur in the Xiannüdong (Stage 3), Mingxinsi (Stage 3), Jindingshan (Stage 4), Tianheban (Stage 4), and, most recently, Guojiaba formations throughout the Yangtze Platform [15,17,18,19,20,21,22,23,24]. Given their global distribution, limited stratigraphic extension, rapid evolution, and exquisite calcareous skeletons, archaeocyaths are crucial to our knowledge on the evolution of metazoans and early Cambrian stratigraphic correlation [15,25,26].

Inverted narrow conical to subcylindrical double-walled cups composed of relatively thick, highly calcified primary elements characterize the morphology of typical archaeocyathan skeletons. They have been interpreted as the extinct taxa of cnidarians (corals) [27,28,29,30,31,32], algae [33,34,35], foraminifera [36], and sponges [28,37,38] based on their morphology, though it is more widely accepted that archaeocyaths are classified as a class of the phylum Porifera and are more closely related to Demospongiae [39]. The filter-feeding structure [40,41] and aquiferous system (water current system) [42] of archaeocyaths are known to have played significant roles. The extraction of archaeocyathan specimens from a limestone matrix with calcite skeletons has proven to be extremely difficult, leading to most studies on archaeocyaths being based solely on thin-section analyses [43]. As a consequence, the study of the morphological structure and systematic classification of the taxon has been significantly limited. Recently, a few three-dimensional phosphatized archaeocyaths have been found [24,44,45,46,47,48,49], which worked as an important supplement to thin-section analysis for the identification of important taxonomical features and development of the cups. Nevertheless, the reports of these fossils mostly focused on their occurrences and taxonomy, lacking comprehensive morphological and anatomical studies. 

In this paper, we present and describe the detailed morphology and anatomy of the three-dimensional phosphatized archaeocyaths from the Shuijingtuo and Xiannüdong formations from South China. It reveals the detailed internal structure and associated water current system, which provides new information for the study of the anatomy, morphological features, and systematic classification of archaeocyaths during the Cambrian period.

## 2. Stratigraphy, Material, and Methods

**Figure 1:**
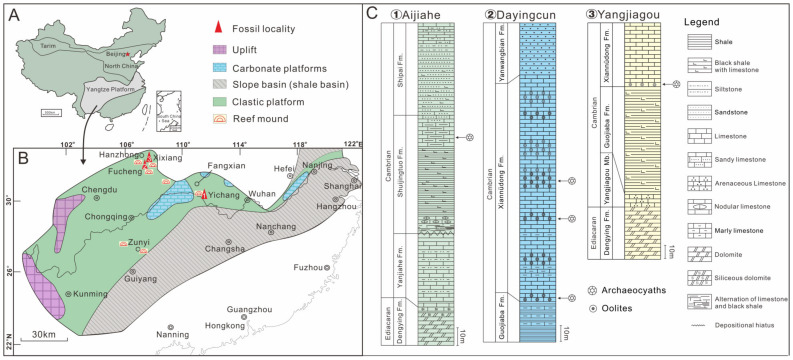
Simplified geological map, paleogeography, and fossil localities. (**A**) Map showing the location of the Yangtze Platform in China. Red star, Beijing. (**B**) A generalized paleogeographic reconstruction of the Yangtze Platform during the early Cambrian period (modified from Zhang et al., 2008 and Zhang et al., 2016 [48,52]); note the relative positions of southern Shaanxi Province and northwestern Hubei Province, marked, respectively, by three numbered solid red triangles. (**C**) Stratigraphic column of the Aijiahe Section, Dayingcun Section, and Yangjiagou Section, showing the distribution of archaeocyathan fossils.

The Yangtze Platform holds significant importance in the investigation of early life evolution due to its well-developed and widely distributed Neoproterozoic to Palaeozoic sedimentary successions [50,51,52]. At the beginning of the Cambrian period, the Yangtze Platform mainly consisted of a siliciclastic platform in the northwest and slope basins in the southeast, with several isolated carbonate platforms scattered among the siliciclastic platform (Figure 1) [48,52]. In particular, carbonate platforms and reefs along the northern margin of the Yangtze Platform typically contain abundant archaeocyaths (Figure 1B). The archaeocyaths described here were derived from the Shuijingtuo and Xiannüdong formations of the Yangtze Platform. Double-walled archaeocyaths with septa were collected from the Shuijingtuo Formation at the Aijiahe Section of Yichang City, western Hubei Province, and the Xiannüdong Formation at the Dayingcun Section of Nanzheng County, southern Shaanxi Province. Double-walled archaeocyaths with canals were collected from the Xiannüdong Formation at the Yangjiagou Section of Xixiang County, southern Shaanxi Province (Figure 1C).

More than 150 samples were collected, including over 100 samples from the Xiannüdong Formation (Dayingcun Section and the Yangjiagou Section) and about 50 samples from the Shuijingtuo Formation (Aijiahe Section). The specimens from limestones were etched in weak acetic acid (5–10%) in the Early Life Institute (ELI), Northwest University, Xi’an, China. The samples were coated with gold and examined further using a scanning electron microscope (SEM FEI Quanta FEG450). Additionally, in order to better investigate the internal structures, three-dimensional reconstructions were conducted through Micro-CT (ZEISS Xradia 520 Versa) (Carl Zeiss, Meditec, Inc., Dublin, OH, USA). Micro-CT images were processed with Dragonfly 4.1 software. The SEM and Micro-CT analyses were conducted at the State Key Laboratory of Continental Dynamics, Northwest University. All fossil specimens were deposited in the ELI.

## 3. Results

### 3.1. Morphology of Archaeocyaths with Septa

**Figure 2:**
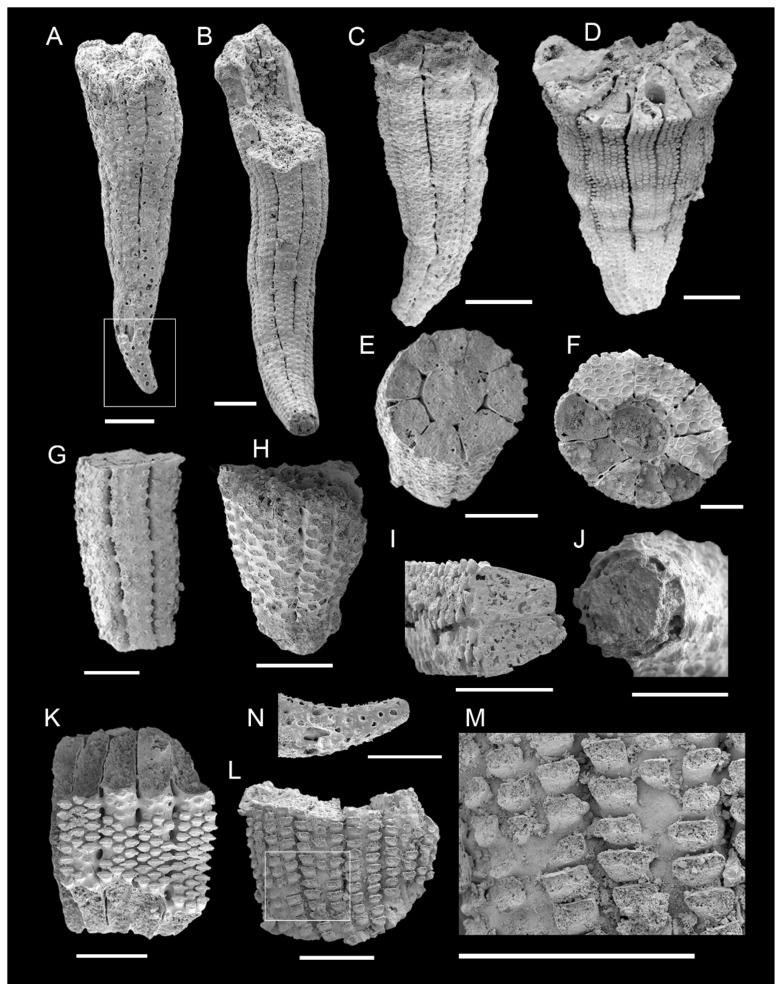
Outer wall morphology of Ajacicyathida from the Shuijingtuo Formation of the Aijiahe Section of Yichang City, western Hubei Province and the Xiannüdong Formation at the Dayingcun Section of Nanzheng County, southern Shaanxi Province. (**A**–**D**) Overall skeletal morphology of Ajacicyathida. (**A**) ELI-AC-040. (**B**) ELI-AC-001. (**C**) ELI-DYC-2-1-02. (**D**) ELI-DYC-2-1-48. (**E**,**F**,**I**) Transverse view showing two walls and septa. (**E**) ELI-AC-006. (**F**) ELI-DYC-2-1-73. (**I**) ELI-AC-030. (**G**,**H**) External view showing a cylindrical shape of incompletely preserved cups. (**G**) ELI-AC-006. (**H**) ELI-AJH-013. (**J**,**N**) The lower part of cup showing the outer wall. (**J**) ELI-AJH-009. (N) ELI-AC-040. (**K**) The pores of outer wall with tumuli. ELI-AC-015. (**L**) The outer wall with perforated elliptical pores. ELI-AJH-015. (**M**) Enlarged view of boxed area in (**L**) showing the addition of the row of pores. ELI-AJH-015. All scale bars = 500 μm.

**Figure 3:**
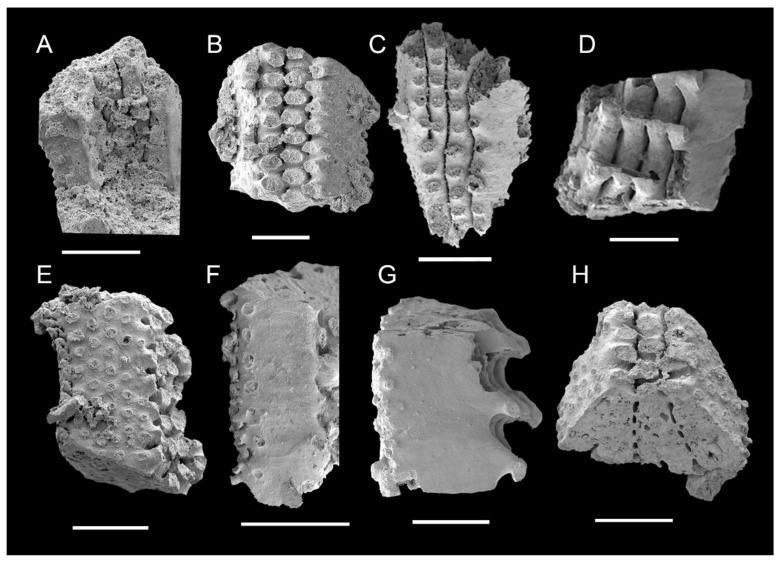
Inner wall and septa morphology of Ajacicyathida from the Shuijingtuo Formation of the Aijiahe Section of Yichang City, western Hubei Province. (**A**–**D**) Longitudinal view of the inner wall with pores. (**A**) ELI-AC-001. (**B**) ELI-AC-025. (**C**) ELI-AJH-005. (**D**) ELI-AC-002. (**E**–**H**) Longitudinal view of the septa with simple round pores. (**E**) ELI-AC-025. (**F**) ELI-AC-030. (**G**) ELI-AC-002. (**H**) ELI-AC-029. All scale bars = 500 μm.

**Figure 4:**
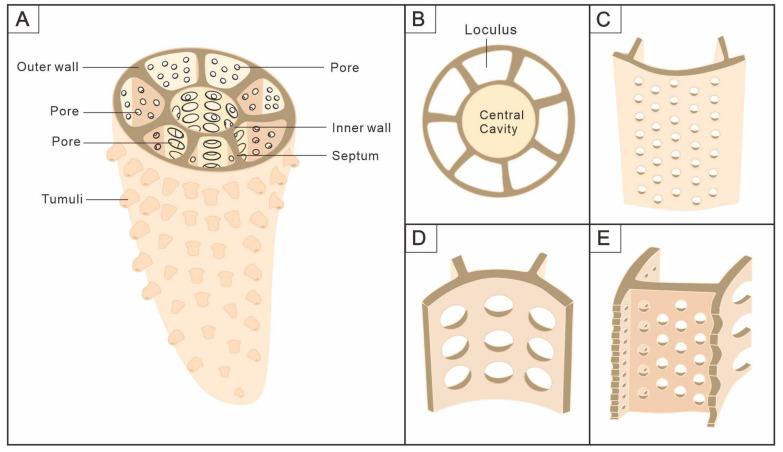
Schematic sketch of Ajacicyathida showing its morphological and anatomical information. (**A**) Morphology of the cup. (**B**–**E**) More detailed features of the main elements. (**B**) Cross-section. (**C**) Outer wall. (**D**) Inner wall. (**E**) Septa.

The cups are narrowly conical in shape (Figure 2A–D), with a maximum height and diameter of 4.58 mm and 1.88 mm, respectively (Table 1). The two inverted porous cones, their inner and outer walls, and the septa that link them make up the primary component of the skeleton that supports the body (Figure 2E,F, Figure 3 and Figure 4). The character of the regular archaeocyaths is consistent with the Order Ajacicyathida [4]. Incomplete cups tend to have a cylindrical shape (Figure 2G,H).

Outer wall—Typically conical in outline. The outer wall is not always smooth but occasionally exhibits various forms of wave-like curvature (Figure 2A–D). Located on the outermost side, the outer wall is perforated by a number of simple pores (Figure 2K–L and Figure 4C) with two to five rows per intersept (Figure 2A–D) and a diameter varying from 0.01 to 0.11 mm (Table 1). In general, the pores are more elliptical toward the top (Figure 2L) and are rounded near the bottom (Figure 2N). Pore rows in the outer wall are typically arranged parallel (Figure 2L) or interspaced (Figure 2K). Several slit-like cracks that divide the outer wall roughly equally are dispersed longitudinally (Figure 2A–D). These cracks are radial longitudinally aligned where the septa join the outer wall, which is the outermost part of the septa. The observation of dozens of fossils corresponding to outer walls indicates that the rows of the outer wall pores between two adjacent septa gradually increase with the cup growth, usually from two to four or five rows (Figure 2B). The newly added rows, with small pore diameters, tend to be inserted from the middle of the two original rows (Figure 2M); nevertheless, as the cup expands, the outer wall pore diameters generally increase. In addition, the morphology of the outer wall pores is not only simple rounded pores, but several cups develop tumuli at the location of the pores (Figure 2K). The majority of cups only have circular pores, which may indicate inadequate preservation.

**Figure 5:**
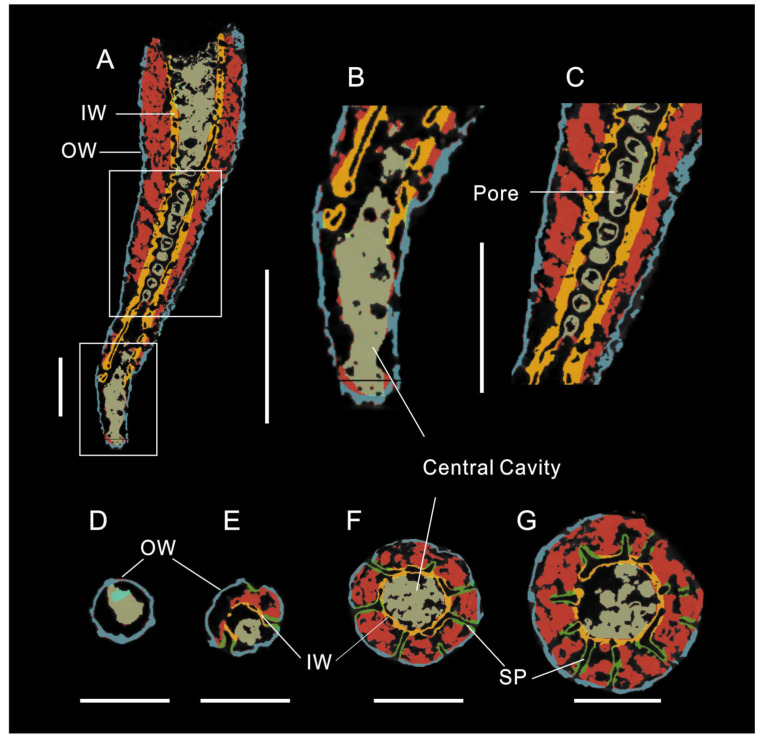
X-ray micro-computer tomography images of Ajacicyathida. ELI-AC-040. (**A**) Anatomical structure of the cup showing two walls with pores, intervallum, and central cavity. (**B**) Enlarged view of lower boxed area in (**A**) showing the lower part of the cup with only outer wall. (**C**) Enlarged view of upper boxed area in (**A**) showing the inner wall pores. (**D**–**G**) The transverse view of cup showing the septa perpendicular to the two walls at different stages. (**D**) Only outer wall at initial stage. (**E**) Two walls and three septa. (**F**) Two walls and seven septa. (**G**) Showing the two walls and ten septa. OW: outer wall; IW: inner wall; SP: septa. All scale bars = 500 μm.

Inner wall—Concentrically distributed with the outer wall (Figure 2E,F). The inner wall only becomes visible when the cup reaches a specific size (about 0.5 mm in height); until then, only the outer wall encloses the central cavity (Figure 5). There are one to two rows of round pores per intersept (Figure 3A–D and Figure 4D) with a diameter of 0.05–0.16 mm in the inner wall (Table 1). The inner wall pores are assigned at intervals (Figure 3A,B) or in parallel (Figure 3C,D) between adjacent septa, and these arrangements can be found in the same specimen. Unlike the outer wall, the increase in the inner wall pore rows is limited by septa. The original intersept split in two when the new septum formed, and the inner wall pores became two smaller rows instead of one row (Figure 3C). The diameter of the inner wall pores subsequently increases in line with the expansion of the intersepta. Moreover, the central cavity is enclosed by the inner wall and typically shown by our specimens to be solidly filled, with a diameter of 0.4 mm (Figure 2E,F). It acts as an exhalant channel, which allows for the circulation of water in the cavity.

The septa—Supporting the body and connecting the inner and outer walls (Figure 4E). The septa are located between two walls and are nearly perpendicular (Figure 2E,F and Figure 5F,G). The septa emerge later than the outer wall and nearly simultaneously with the inner wall (Figure 5B,E), and the number of septa grow as the diameter of the cup increases (Figure 5E–G). A cup commonly contains 9 septa on average and 12 septa as a maximum (Table 1). Either the septum is uniformly perforated (Figure 3E,H) with up to 7–8 rows of pores, or the pores are dispersed with as few as 1 row on the side closest to the outer wall (Figure 3F,G). The pore rows are normally staggered (Figure 3E) and have a diameter of 0.07 mm on average, which is smaller than the pores of the inner wall but larger than the outer wall (Table 1). The intervallum (the area between the two walls) is divided by septa into similar-sized chambers, called loculus (Figure 4B), with a width of 0.31 mm (Table 1), and each of them is isolated from the others and connected by septa pores. The loculi could be the areas where the soft tissue is located [53]. The living tissue may have decayed after burial and the loculi are filled.

Tabulae–Transverse plates known as tabulae, which link the two walls perpendicular to the septa, are porous and flat to slightly arched (Figure 2F). Nearly the entire tabulae are covered by circular pores with a diameter of 0.09 mm (Table 1), indicating a high porosity. The pore size is larger than the septa but similar to the inner wall (Table 1). The pores are successively distributed among the tabulae, which are joined to the inner walls by a smooth arch with no truncated angular gap (Figure 2F). The presence of tabulae may characterize different types of the cup [4]. Transverse plates are not common in some fossils from earlier stages of development and may indicate that the fossil developed to a certain stage [4,54].

### 3.2. Morphology of Archaeocyaths with Canals

**Figure 6:**
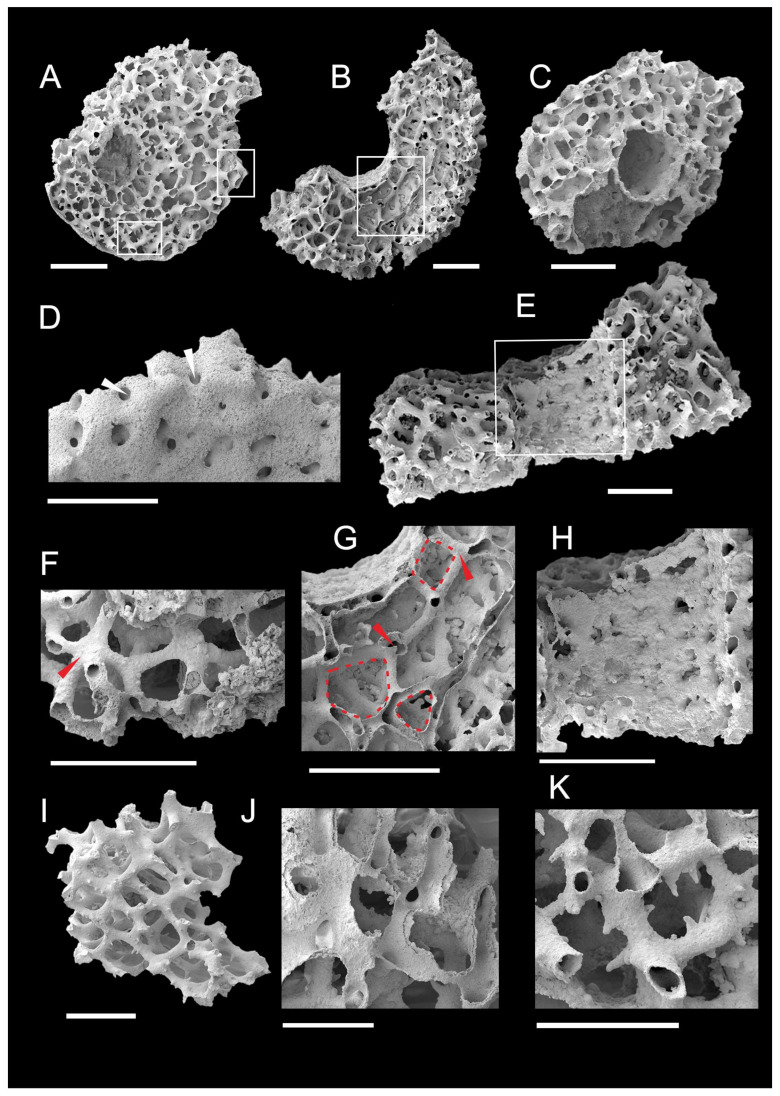
Archaeocyaths from the Xiannüdong Formation at the Yangjiagou Section of Xixiang County, southern Shaanxi Province. (**A**–**C**) Transverse view showing the two walls and hollow canals. (**A**) ELI-XX-XND-1-12. (**B**) ELI-XX-XND-1-07. (**C**) ELI-XX-XND-1-14. (**D**) Enlarged view of right boxed area in (**A**) showing pores of the outer wall (marked by white arrows). ELI-XX-XND-1-12. (**E**,**H**) Longitudinal view showing the inner wall. ELI-XX-XND-1-07. (**E**) Inner wall and canals of intervallum. (**H**) Enlarged view of boxed area in (**E**) pores of the inner wall. (**F**,**G**) The details of canals. (**F**) Convergence of five canals (marked by red arrows). ELI-XX-XND-1-22. (**G**) Enlarged view of boxed area in (**B**) showing convergence of three and four canals (marked by red arrows), and triangular, quadrilateral, and pentagonal shapes of cavities enclosed by canals (marked by red dashed line). ELI-XX-XND-1-07. (**I**) Fossil fragments showing solid canals. ELI-XX-XND-1-25. (**J**) Broken canals showing the two walls of canals. ELI-XX-XND-1-03. (**K**) The enlarged view of lower boxed area in (**A**) showing centripetal distribution of small spines on the canals. Scale bars represent 500 μm (**A**–**J**); 250 μm (**K**).

The cups lack a distinct columnar morphology and exhibit a flattened, low cylindrical shape with an occasionally off-center inner wall (Figure 6A). There are canal structures instead of septa connecting the inner and outer walls, showing transverse and longitudinal networks (Figure 6A–C). Incomplete cups show only the canals (Figure 6I). 

The cylindrical outer wall functions as a supportive skeleton to provide protection to the soft body. The outer wall has a series of pores (Figure 6D), with a diameter of 0.043 mm. The perforations facilitate communication between the external environment and the cavity in the intervallum. The membrane that secretes the outer wall is preserved and wrapped around the canal structure that lies between the walls (Figure 6A). The outer wall interconnects with the canals and inward to the inner wall to collectively establish the skeletal structure of the cup.

The inner and outer walls are distributed concentrically, surrounding a central cavity with a diameter of 0.9 mm (Table 2). The pores in the wall (Figure 6E) are a significant component of the exhalant system, connecting the cavity in the intervallum. The complete inner wall has approximately eight to eleven rows of pores averaging 0.08 mm in diameter (Table 2) that are arranged in parallel (Figure 6H).

Canals, as the intervallum component connecting the inner and outer walls, possess parallel and perpendicular morphologies to the two walls, respectively (see Supplementary Materials Videos S1 and S2). The canals are intricately interconnected, exhibiting a general inward curvature similar to that of the inner and outer walls (Figure 6A–C). The diameter of the canals is usually 0.06 mm (Table 2), with larger diameters closer to the intersections (Figure 6F). There are three primary types of intersections where horizontal and vertical canals converge, specifically three, four, and five canals of intersections (Figure 6F,G). Typically, three canals of intersections, where all three canals are almost in the same plane (Figure 6G); four canals of intersections, where three are in the same plane and the fourth is perpendicular to them (Figure 6G); five canals of intersections, where four are in the same plane and the fifth is perpendicular to them, or three of them aligned in a plane and the other two are perpendicular to it (Figure 6F). The inter-spaces among canals are mostly triangular, quadrilateral, and pentagonal in the transverse section (Figure 6G). The canals are arranged as solid (Figure 6I) and hollow (Figure 6A–C), with some fossils showing two layers (Figure 6J) and hollow canals potentially retaining an outer membrane that is 0.008 mm thick (Table 2). Solid canals are essentially the result of hollow canals being filled. Furthermore, a few canals contain tiny spines that are 0.026 mm in diameter and 0.033 mm in length (Table 2), growing centripetally on the outside (Figure 6K).

## 4. Discussion

### 4.1. Comparison of the Two Archaeocyaths

There are two main distinct types of phosphatized archaeocyaths that we have described; one is the type with radiating septa and the other is with canals between the walls. They are similar in that both have perforated inner and outer walls, and the central cavity is surrounded by the inner wall. However, the former two-walled cup is clearly narrower and more conical in shape, with septa in the intervallum, and may have plate tabulae, which are typically characteristic of the Order Ajacicyathida [55]. The latter shows a low and columnar cup with double walls and canals in the intervallum. It is worth mentioning that these phosphatized archaeocyaths with canals are found for the first time and lack sufficient material for detailed comparison. Therefore, there remain some difficulties in their identification and classification. Morphologically, the canals are not limited to a sole plane and share similarities with taeniae, pseudotaenial, or dictyonal networks of the Order Archaeocyathida [4,55,56]. A dictyonal network comprises synapticulae and taenial lintels, together forming an orthogonal network of rods [4,56]. Nevertheless, our material demonstrates that the network is composed of canals rather than a structure resembling synapticulae. The definitions of a pseudotaenial or dictyonal network are given based on thin-section analysis, which may have some obstacles in revealing their actual morphology [23].

**Figure 7:**
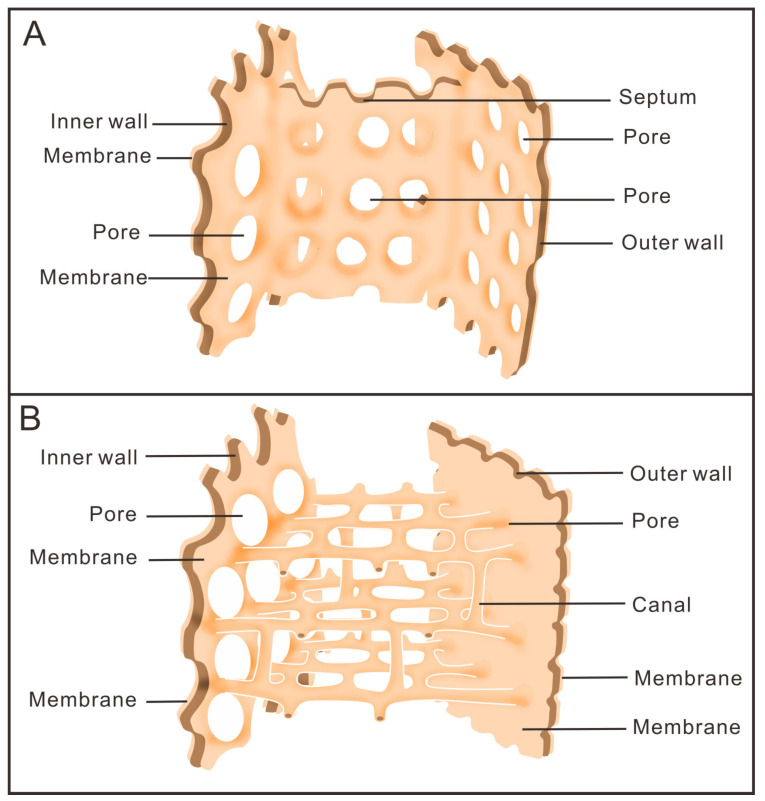
Schematic reconstruction of anatomical structure between outer wall and inner wall of archaeocyaths. They show that the skeletons that support the cup are secreted by the membrane that surrounds them. (**A**) Archaeocyaths with septa, showing the septa connecting the inner and outer walls, and the pores in them making the inside and outside of the cup accessible. (**B**) Archaeocyaths with canals, showing the canals connecting the inner and outer walls, the pores in the two walls, and the canals connected for internal and external communication.

On the other hand, these two types exhibit a distinctive preservation. The cups with septa are preserved as the internal mold and it is assumed that the soft tissue is confined to the loculi [41,53]. Whilst the cups with canals preserve the membranes that may secrete their skeletons, which were first reported in South China, although similar materials, identified as ajacicyathids, have been found in Siberia [57]. The walls and septa, important components of the skeleton, have been etched away by acid, leaving only the membrane that covers the exterior. The fact that the two different types of cups appear to have the same preservation suggests that the skeleton of the cups is derived from membrane secretion, with the structure of the intervallum being the primary difference between them (Figure 7). As filter feeders, these two distinct types of structure between the two walls may imply a slight variation in the water current system. But the process of flow remains the same: outer currents enter the loculi through the outer wall pores and leave through the inner wall pores [4,42]. 

### 4.2. Comparison with Coral

Based on the shape of their calcareous skeletons and the presence of septa, archaeocyaths used to be recognized as corals [27,28,29,30,31]. It was widely accepted by the end of the 19th century that archaeocyaths are a distinctive group of pore-bearing corals [32,39]. There are many similarities found in archaeocyaths and corals. Regarding the overall morphology, the basic shapes of both solitary corallum and archaeocyaths are curved or erect inverted cones [4,58]. The mural pores in many tabulate corals, and varying degrees of porosity in some rugose corals and many scleractinian corals, morphologically closely resemble archaeocyaths with perforated calcareous skeletons [39,58]. In addition, most solitary corallum have evolved an outer wall that extends to the edge of the calice. The walls may be extended as hollow or with solid spines, which are known as connecting tubus in fasciculate coralla to fix a corallite to its neighbors [58]. Similarly, archaeocyaths produced abundant secondary skeletal linkages between adjacent cups, such as the tumuli (Figure 4A), which may have facilitated in communication between the cups of the colony [4,59]. However, there are some obvious differences between the two taxa. Corals do not always possess inner walls, and even those that do display mostly discontinuous internal structures. In contrast, most archaeocyaths have the well-defined and distinctive inner wall [58,60]. Furthermore, despite the fact that they both have septa, individual corallum of Rugosa typically exhibit septa radiating from the corallite center, with bilateral symmetry indicated by the mode of septal insertion [58,61]. Nonetheless, the insertion of the septa of the cup is based on the width of the loculi in the cross-section. The loculus width is maintained by the insertion of new septa once the loculus width reaches a certain size (Figure 5F,G) [4,62], albeit the specific insertion mode is unknown. The living tissue associated with feeding in archaeocyaths is confined to the loculi, which is also quite different from that of corals [41,42]. The polyp of rugose coral is assumed to be similar in structure to other cnidarian polyps, with rings of tentacles surrounding the opening at the top that functions both as a mouth and an anus, and for reproduction [58]. Archaeocyaths, on the other hand, are likely to have filter-feeding structures that filtered water currents through wall pores to obtain nutrients [40,41,42].

### 4.3. Comparison with Sponge

Archaeocyaths were widely recognized as an extinct class of Porifera based on the microstructure and functional morphology [41,53,63,64,65,66]. For the basic morphological structure, the body wall of sponges consists of two layers of cells, which are the dermal epithelium and the gastral epithelium (choanocyte layer), and between these two, there is the non-cellular structure of the mesoglea, which contains the amoebocytes and spicules [67]. On the contrary, archaeocyaths lack spicules or amoebocytes, and are mainly composed of the inner and outer walls, as well as the structures between the walls (Figure 4). Obviously, the morphology of archaeocyaths is radically different from that of any living sponges, with the exception of the genus *Vaceletia* [68,69], which is the extant aspiculate hypercalcified demosponge and is strikingly similar to the thalamid chambered archaeocyaths (order Kazachstanicyathida) in growth habit [70,71,72]. There are several similarities between *Vaceletia*, the only surviving sphinctozoan sponge, and the archaeocyaths, including the absence of spicules, the massive calcareous skeleton, and the segmentation and partitioning of the skeleton [65]. Furthermore, it is remarkable that *Celyphia norica*, a species of single-chambered sphinctozoans from the Upper Triassic of Sicily, possesses structures similar to septa [73]. Nonetheless, some fundamental distinctions exist, such as the elaboration of wall pores in archaeocyaths, which is completely lacking in sphinctozoans. And their ontogenetic sequence of wall formation and wall microstructures are different, especially in regard to the intervallum, where food processing takes place [65]. Moreover, another non-spicular sponge has a skeleton composed of fibrous networks with a regular shape and consistent thickness [74], and these fibers are clearly round or elliptical in the transverse section, resembling the canals of archaeocyaths (Figure 6). Nevertheless, they serve different functions throughout the life of organisms.

Traditionally, the existence of an aquiferous system (water current system) is another factor that provides support for the view that archaeocyaths belonged to sponges [42]. Generally, the aquiferous system of sponges consists mainly of pores, and the ostium lined by pinacocytes that allows for water to enter or exit the choanocyte chambers. The choanocytes that line these chambers are in charge of moving the water through the system [75]. The unique porous double-walled structure of the cups was admirably suited to filtration in ambient flow. Crucial components of the exhalant and the inhalant systems, respectively, are the inner and outer wall pores (Figure 2 and Figure 3). Unlike the mesoglea of sponges, the living tissue is confined to the loculi and consists of generalized cells grouped in specialized associations in individual loculi (Figure 4A,B) [54,76,77,78,79]. Via the pores of the outer wall, fluid moves into the loculi where the living tissue absorbs nutrients from the stream of water and, finally, leaves through the inner wall pores at the top of the central cavity [41]. The choanocyte chambers have only been demonstrated to exist in the loculi or cavities between the two walls of archaeocyaths indirectly; however, there is no definitive evidence [4,64].

Astrorhizae, the star-like canal structures in various hypercalcified sponges, have also been discovered in several archaeocyaths [4,80]. Identification of astrorhizae is considered a distinctive feature of choanocyte-bearing organisms [80,81]. It is plausible that some archaeocyaths possessed astrorhizal canals that were embedded in the soft tissue, comparable to some living demosponges with non-spiculate skeletons [4]. Although having similar canal structures, the canals in the cup are arranged more regularly than astrorhizae (Figure 6). Astrorhizae have been widely accepted as constructs associated with aquiferous systems (water current system) in organisms [82], but their functional and biological properties are still not clearly defined. Moreover, the evidence is currently insufficient because of the limited discovery of astrorhizae in archaeocyaths and restricted thin-section analysis [55].

Furthermore, in contrast to the conventional theory on water filtration mechanisms in archaeocyaths, cups with canals exhibit structural intricacy which possibly suggesting the evolutionary appearance of more efficient water feeding of archaeocyaths. There also exists speculation that these canals may represent calcified metazoan internal plumbing features, possibly resembling the water vascular system in echinoderms [83], such as Figure 17 refer to Ref. [84]. The canal-bearing fossils (Figure 6) may also represent the “missing link” between typical archaeocyaths and metazoans. The hollow canals provide evidence favoring a hypothesis about archaeocyath affinities that the archaeocyaths may represent an “archaeozoan” grade of organization that is intermediate between the metazoan and parazoan grade [85,86]. Nevertheless, more well-preserved material and more advanced techniques should be employed to further examine the affinity of these intriguing and important taxa. 

## 5. Conclusions

Here, phosphatized early Cambrian archaeocyaths from the Shuijingtuo Formation (Stage 3) and Xiannüdong Formation (Stage 3) of the Yangtze Platform are reported, and their morphological and anatomical structures are described. The results provide two distinct types of archaeocyaths. Cups with septa basically belong to the Order Ajacicyathida of regular taxon, with perforated inner walls, outer walls, and septa, which are exquisitely preserved. And cups with well-defined complicated interior network canals connecting the inner and outer walls and the pores work as an important part of the water current system and influence the filter-feeding structure and efficiency of the cup. Intricate canals also suggest that archaeocyaths may have a closer relationship with metazoans than with sponges. The combination of SEM and Micro-CT reconstruction fills gaps in the analysis of thin sections in the study of Cambrian archaeocyaths and contributes to the finely microscopic morphological structure, which provides important clues for exploring the systematic taxonomic, anatomical, and morphological characteristics of Cambrian archaeocyaths.

## Figures and Tables

**Table 1:** Measurements of Ajacicyathida.

	H	D(C)	Int	IS	N	RK	IK	IC	D (OWP)	D (IWP)	D (SPP)	D (TAP)
Count	27	52	54	61	54	21	23	54	103	48	65	4
Min	1.04	0.59	0.1	0.1	6	8.38	0.19	0.29	0.01	0.05	0.03	0.06
Max	4.58	1.88	0.57	0.48	12	17.19	0.32	1.4	0.11	0.16	0.1	0.14
Mean	2.04	1.06	0.31	0.24	9	11.74	0.28	0.81	0.05	0.1	0.07	0.09
SD	0.6	0.34	0.11	0.07	1	2.95	0.026	0.21	0.03	0.03	0.018	0.015

H, cup height; D, diameter; Int, intervallum width (the width of loculus); IS, intersepta; N, number of septa; RK, radial coefficient = number of septa/cup diameter; IK = Int/D; IC = IS/Int: ratio of sides of loculi in transverse section; C, cup; OWP, outer wall pore; IWP, inner wall pore; SPP, septa pore; TAP, tabulae pore. SD, standard deviation. All measurements are in millimeter.

**Table 2:** Measurements of Archaeocyaths with canals.

	D(C)	Int	IK	D (CA)	T (CA)	D (OWP)	D (IWP)	D (SPI)	L (SPI)
Count	3	2	2	26	20	5	3	3	3
Min	1.47	0.46	0.23	0.02	0.003	0.029	0.05	0.015	0.02
Max	2.01	0.95	0.58	0.137	0.018	0.112	0.11	0.035	0.04
Mean	1.82	0.72	0.38	0.06	0.008	0.056	0.08	0.026	0.03
SD	0.16	0.18	0.13	0.015	0.003	0.022	0.018	0.005	0.006

D, diameter; Int, intervallum width; IK = Int/D**(C)**; T, thickness; L, length; C, cup; CA, canal; OWP, outer wall pore; IWP, inner wall pore; SPI, spine. SD, standard deviation. All measurements are in millimeter.

## Data Availability

The data presented in this study are available in this article and Supplementary Materials.

## Supplementary Materials

The following supporting information can be downloaded at: https://www.mdpi.com/article/10.3390/life14020167/s1, Video S1: 3D visualization of archaeocyaths with canals (ELI-XX-XND-1-12); Video S2: 3D visualization of archaeocyaths with canals (ELI-XX-XND-1-14).

## References

[1] Sepkoski J.J. (1979). A kinetic model of Phanerozoic taxonomic diversity II: Early Phanerozoic families and multiple equilibria. Paleobiology.

[2] James N.P., Debrenne F. (1980). Lower Cambrian bioherms: Pioneer reefs of the Phanerozoic. Acta Palaeontol. Pol..

[3] Zhang X.L., Shu D.G., Han J., Zhang Z.F., Liu J.N., Fu D.J. (2014). Triggers for the Cambrian explosion: Hypotheses and problems. Gondwana Res..

[4] Debrenne F., Zhuravlev A.Y., Kruse P.D., Stearn C.W. (2015). General features of Archaeocyatha. Treatise on Invertebrate Paleontology.

[5] Shu D.G., Han J. (2020). The core value of Chengjiang fauna: The formation of animal kingdom and the birth of basic human organs. Earth Sci. Front..

[6] Hanfield R.C. (1971). Archaeocyatha from the Mackenzie and Cassiar Mountains, Northwest Territories, Yukon Territory and British Columbia. Geol. Surv. Can. Bull..

[7] Gravestock D.I. (1984). Archaeocyatha from lower parts of the Lower Cambrian carbonate sequence in South Australia. Mem. Assoc. Australas. Paleontol..

[8] Debrenne F., Kruse P.D. (1986). Shackleton limestone archaeocyaths. Alcheringa.

[9] Zhang J.M., Yuan K.X. (1994). Archaeocyath reefs from Lower Cambrian Tianheban Formation at Wangjiaping, Yichang, Hubei and their diagenesis. Chin. J. Geol..

[10] Kruse P.D., Zhuravlev A.Y., James N.P. (1995). Primordial metazoan-calcimicrobial reefs: Tommotian (Early Cambrian) of the Siberian platform. Palaios.

[11] Riding R., Zhuravlev A.Y. (1995). Structure and diversity of oldest sponge-microbe reefs: Lower Cambrian, Aldan River, Siberia. Geology.

[12] Gandin A., Debrenne F. (2010). Distribution of the archaeocyath-calcimicrobial bioconstructions on the Early Cambrian shelves. Palaeoworld.

[13] Menéndez S., Rodríguez-Martínez M., Moreno-Eris E., Perejón A., Reitner J. (2010). Palaeoenvironmental and geochemical approach of Archaeocyath-rich facies from Lower Cambrian of Western Gondwana margin at Central Iberian Zone (Urda, Toledo Mountains, Spain). Geophys. Res. Abstr..

[14] Stone P., Thomson M.R.A., Rushton W.A. (2011). An Early Cambrian archaeocyath–trilobite fauna in limestone erratics from the Upper Carboniferous Fitzroy Tillite Formation, Falkland Islands. Earth Environ. Sci. Trans. R. Soc. Edinb..

[15] Yang A.H., Zhu M.Y., Zhuravlev A.Y., Yuan K.X., Zhang J.M., Chen Y.Q. (2016). Archaeocyathan zonation of the Yangtze Platform: Implications for regional and global correlation of lower Cambrian stages. Geol. Mag..

[16] Rodriguez-Martinez M., Buggisch W., Menendez S., Moreno-Eiris E., Perejón A. (2022). Reconstruction of a Ross lost Cambrian Series 2 mixed siliciclastic–carbonate platform from carbonate clasts of the Shackleton Range, Antarctica. Earth Environ. Sci. Trans. R. Soc. Edinb..

[17] Yuan K.X., Zhang S.G., Three Yangtze Gorges Geological Research Unit, Geological Bureau of Hubei Province (1978). Archaeocyatha. Stratigraphy and Palaeontology of Sinian to Permian in the Eastern Part of the Yangtze Gorge.

[18] Yuan K.X., Zhang S.G. (1980). Lower Cambrian Archaeocyatha of central and southwestern China. Acta Palaeontol. Pol..

[19] Yuan K.X., Zhang S.G. (1981). Lower Cambrian archaeocyathid assemblages of central and southwestern China. Spec. Pap. Geol. Soc. Am..

[20] Yuan K.X., Zhang S.G. (1983). Biogeographical provinces of Early Cambrian archaeocyathids in China. Nanjing Inst. Geol. Palaeontol. Acad. Sin. Bull..

[21] Zhang S.G., Yuan K.X. (1984). Lower Cambrian archaeocyathids in Weiganping from Fuquan, China. Acta Palaeontol. Sin..

[22] Yuan K.X., Zhu M.Y., Zhang J.M., Vaniten H. (2001). Biostratigraphy of archaeocyathan horizons in the lower Cambrian Fucheng section, South Shaanxi Province: Implications for regional correlations and archaeocyathan evolution. Acta Palaeontol. Sin..

[23] Yang A.H., Yuan K.X. (2012). New archaeocyaths from the early Cambrian of Shaanxi and Guizhou provinces, South China. Geobios.

[24] Luo M., Liu F., Liang Y., Strotz L.C., Wang J., Hu Y., Song B., Holmer L.E., Zhang Z. (2023). First Report of Small Skeletal Fossils from the Upper Guojiaba Formation (Series 2, Cambrian), Southern Shaanxi, South China. Biology.

[25] Rozanov A.Y., Missarzhevskiy V.V., Volkova N.A., Voronova L.G., Krylov I.N., Keller B.M., Korolyuk I.K., Lendzion K., Mikhnyak R., Pykhova N.G. (1969). The Tommotian Stage and the Cambrian lower boundary problem. Trans. Acad. Sci. USSR Nauka.

[26] Zhuravlev A.Y. (2015). The early history of the Metazoa—A paleontologist’s viewpoint. Biol. Bull. Rev..

[27] Bayfield (1845). On the junction of the transition and primary rocks of Canada and Labrador. Q. J. Geol. Soc. Lond..

[28] Billings E. (1861). New Species of Lower Silurian Fossils.

[29] Meek F.B. (1868). Preliminary notice of a remarkable new genus of corals, probably typical of a new family. Am. J. Sci..

[30] Bornemann J.G. (1886). Die Versteinerungen Des Cambrischen Schichtensystems Der Insel Sardinien Nebst Vergleichenden Untersuchungen über Analoge Vorkommnisse Aus Andern Ländern.

[31] Hinde G.J. (1889). On Archaeocyathus, Billings, and on other Genera, allied to or associated with it, from the Cambrian Strata of North America, Spain, Sardinia, and Scotland. J. Geol. Soc..

[32] Walcott C.D. (1894). The Fauna of the Lower Cambrian or Olenellus Zone.

[33] Toll E. (1899). Beiträge zur Kenntniss des Sibirschen Cambrium. Imp. Acad. Sci. St. Petersburg Mem..

[34] Öpik A.A. (1975). Cymbric Vale fauna of New South Wales and Early Cambrian biostratigraphy. Aust. Gov. Publ. Serv. Bur. Miner. Resour. Geol. Geophys. Bull..

[35] Nitecki M.H., Toomey D.F. (1979). Nature and classification of receptaculitids. Cent. Rech. Explor.-Prod. Elf-Aquitaine.

[36] Meek F.B. (1868). Note on Ethmophyllum and Archaeocyathus. Am. J. Sci. 2d Ser..

[37] Billings E. (1865). Paleozoic Fossils.

[38] Walcott C.D. (1886). Cambrian faunas of North America. U. S. Geol. Surv. Bull..

[39] Rowland S.M. (2001). Archaeocyaths—A history of phylogenetic interpretation. J. Paleontol..

[40] Balsam W.L., Vogel S. (1973). Water movement in archaeocyathids: Evidence and implications of passive flow in models. J. Paleontol..

[41] Savarese M. (1992). Functional analysis of archaeocyathan skeletal morphology and its paleobiological implications. Paleobiology.

[42] Zhuravlev A.Y. (1993). A functional morphological approach to the biology of the Archaeocyatha. Neues Jahrb. Geol. Paläontol. Abh..

[43] Rigby J.K., Gangloff R.A., Boardman R.S., Cheetham A.H., Rowell A.J. (1987). Phylum Archaeocyatha. Fossil Invertebrates.

[44] Dzik J. (1994). Evolution of ‘small shelly fossils’ assemblages of the Early Paleozoic. Acta Palaeontol. Pol..

[45] Wrona R. (2004). Cambrian microfossils from glacial erratics of King George Island, Antarctica. Acta Palaeontol. Pol..

[46] Skovsted C.B. (2006). Small Shelly fauna from the upper Lower Cambrian Bastion and Ella Island formations, north-east Greenland. J. Paleontol..

[47] Smith E.F., Macdonald F.A., Petach T.A., Bold U., Schrag D.P. (2016). Integrated stratigraphic, geochemical, and paleontological late Ediacaran to early Cambrian records from southwestern Mongolia. Geol. Soc. Am. Bull..

[48] Zhang Z.F., Zhang Z.L., Li G.X., Holmer L.E. (2016). The Cambrian brachiopod fauna from the first-trilobite age Shuijingtuo Formation in the Three Gorges area of China. Paleoworld.

[49] Pruss S.B., Dwyer C.H., Smith E.F., Macdonald F.A., Tosca N.J. (2019). Phosphatized early Cambrian archaeocyaths and small shelly fossils (SSFs) of southwestern Mongolia. Palaeogeogr. Palaeoclimatol. Palaeoecol..

[50] Wang X.F., Erdtmann B.D., Chen X.H., Mao X.D. (1998). Integrated sequence-, bio- and chemostratigraphy of the terminal Proterozoic to Lowermost Cambrian. Epis. J. Int. Geosci..

[51] Zhu M., Zhang J., Yang A. (2007). Integrated Ediacaran (Sinian) chronostratigraphy of South China. Palaeogeogr. Palaeoclimatol. Palaeoecol..

[52] Zhang Z., Robson S.P., Emig C., Shu D. (2008). Early Cambrian radiation of brachiopods: A perspective from South China. Gondwana Res..

[53] Wood R.A., Zhuravlev A.Y., Debrenne F. (1992). Functional biology and ecology of Archaeocyatha. Palaios.

[54] Zhuravleva I.T. (1959). O polozhenii arkheotsiat v filogeneticheskoy sisteme [On the position of archaeocyaths in the phylogenetic system]. Paleontol. Zhurnal.

[55] Debrenne F., Zhuravlev A.Y., Kruse P.D., Selden P.A., Stearn C.W. (2015). Systematic descriptions: Archaeocyatha. Treatise on Invertebrate Paleontology.

[56] Debrenne F., Zhuravlev A.Y. (1992). Irregular Archaeocyaths: Morphology, Ontogeny, Systematics, Biostratigraphy, Palaeoecology.

[57] Kouchinsky A., Alexander R., Bengtson S., Bowyer F., Clausen S., Holmer L.E., Zhuravlev A.Y. (2022). Early-middle Cambrian stratigraphy and faunas from northern Siberia. Acta Palaeontol. Pol..

[58] Hill D., Robison R.A. (1981). Coelenterata. Rugosa and Tabulata. Part F. Supplement 1. Treatise on Invertebrate Paleontology.

[59] Debrenne F., Reitner J., Zhuravlev A., Riding R. (2000). Sponges, cnidarians, and ctenophores. The Ecology of the Cambrian Radiation.

[60] Taylor T.G. (1910). The Archaeocyathinæ from the Cambrian of South Australia: With an account of the morphology and affinities of the whole class. Mem. Soc. S. Aust..

[61] Cohen A.L., McConnaughey T.A. (2003). Geochemical perspectives on coral mineralization. Rev. Mineral. Geochem..

[62] McKee E.H. (1963). Ontogenetic stages of the archaeocyathid *Ethmophyllum whitneyi* Meek. J. Paleontol..

[63] Kruse P.D., Debrenne F. (1989). Review of archaeocyath microstructure. Mem. Assoc. Australas. Palaeontol..

[64] Zhuravlev A.Y. (1989). Poriferan aspects of archaeocyathan skeletal function. Mem. Assoc. Australas. Palaeontol..

[65] Kruse P.D. (1990). Are archaeocyaths sponges, or are sponges archaeocyaths. Aust. Geol. Soc. Spec. Publ..

[66] Wood R.A. (1990). Reefbuilding sponges. Am. Sci..

[67] Leys S.P., Hill A. (2012). The physiology and molecular biology of sponge tissues. Adv. Mar. Biol..

[68] Vacelet J. (1977). Une nouvelle relique du Secondaire: Un représentant actuel des éponges fossiles Sphinctozoaires. Comptes Rendus Acad. Sci..

[69] Wörheide G., Reitner J., Reitner J., Neuweiler F., Gunkel F. (1996). “Living fossil” sphinctozoan coralline sponge colonies in shallow water caves of the Osprey Reef (Coral Sea) and the Astrolabe Reefs (Fiji Islands). Global and Regional Controls on Biogenic Sedimentation, Vol. 1. Reef Evolution.

[70] Debrenne F., Vacelet J. (1984). Archaeocyatha: Is the sponge model consistent with their structural organization. Palaeontogr. Am..

[71] Pickett J. (1985). Vaceletia, a living archaeocyathid. N. Z. Geol. Surv. Rec..

[72] Reitner J., Wörheide G., Lange R., Thiel V. (1997). Biomineralization of calcified skeletons in three Pacific coralline demosponges-an approach to the evolution of basal skeletons. Cour. Forschungsinstitut Senckenberg.

[73] Senowbari-Daryan B., Schäfer P. (1986). Sphinctozoen (Kalkschwämme) aus den norischen Riffen von Sizilien. Facies.

[74] Luo C., Reitner J. (2014). First report of fossil “keratose” demosponges in Phanerozoic carbonates: Preservation and 3-D reconstruction. Sci. Nat..

[75] Simpson T.L. (1984). The Cell Biology of Sponges.

[76] Zhuravleva I.T. (1963). Arkheotsiaty Sibiri: Odnostennye arkheotsiaty: Otriady Monocyathida i Rhizacyathida.

[77] Zhuravleva I.T. (1974). Biologiya arkheotsiat [Biology of archaeocyaths]. Tr. Inst. Geol. Geofiz. Sib. Otd..

[78] Zhuravleva I.T., Elkina V.N. (1974). Arkheotsiaty Sibiri: Etmophilloidnye Arkheotsiaty [Arqueociatos de Siberia: Arqueociatos ethmofiloides]. Tr. Inst. Geol. Geofiz. Sib. Otd. Akad. Nauk SSSR.

[79] Zhuravleva I.T., EI M. (1979). Comparaison entre les Archaeata et les Porifera. Colloq. Int. CNRS.

[80] Boyajian G.E., LaBarbera M. (1987). Biomechanical analysis of passive flow of stromatoporoids—Morphologic, paleoecologic, and systematic implications. Lethaia.

[81] Hartman W.D. (1983). Modern and ancient sclerospongiae. Ser. Geol. Notes Short Course.

[82] Stearn C.W. (1975). The stromatoporoid animal. Lethaia.

[83] Briggs D.E., Siveter D.J., Siveter D.J., Sutton M.D., Rahman I.A. (2017). An edrioasteroid from the Silurian Herefordshire Lagerstätte of England reveals the nature of the water vascular system in an extinct echinoderm. Proc. R. Soc. B..

[84] Peel J.S., Streng M., Geyer G., Kouchinsky A., Skovsted C.B. (2016). ‘*Ovatoryctocara granulate*’ assemblage (Cambrian series 2-series 3 boundary) of Londal, North Greenland. Australas. Palaeontol. Mem..

[85] McMenamin M.A.S. (2016). Dynamic Paleontology.

[86] McMenamin M.A.S. (2023). The Cambrian Explosion: Macroevolution and biomineralization. Acad. Biol..

